# Utilizing non-stationary extreme value model to quantify extreme rainfall in two major cities in Bangladesh

**DOI:** 10.1007/s00477-025-02969-3

**Published:** 2025-04-07

**Authors:** Asim K. Dey, Mohammad Shaha A. Patwary

**Affiliations:** 1 Department of Mathematics and Statistics, Texas Tech University, 1108 Memorial Circle, Lubbock, 79409 Texas USA; 2 Mathematical Sciences, Butler University, 4600 Sunset Avenue, Indianapolis, 46208 Indiana USA

**Keywords:** Extreme rainfall, Block maxima approach, Return level, Probability of extreme, Bootstrap confidence interval

## Abstract

Bangladesh is highly susceptible to the impacts of climate change, particularly extreme rainfall during the monsoon season, leading to severe floods and landslides. This study introduces a nonstationary generalized extreme value (GEV) modeling framework, which integrates atmospheric dry bulb temperatures as a covariate to capture the seasonal and dynamic characteristics of extreme rainfall events. Using daily rainfall and temperature data from Dhaka (1990–2015) and Chattogram (1999–2015), the study identifies optimal models based on AIC, BIC, and goodness-of-fit criteria. Uncertainties in the predictions are quantified using the delta method and parametric bootstrap approaches. The results indicate a higher likelihood of extreme rainfall events in Chattogram compared to Dhaka, as reflected in the predictions and probabilities in return levels. Diagnostic evaluations confirm that the models effectively capture the variability in monthly maximum rainfall during the monsoon. These findings offer valuable information for flood risk management, urban planning, and disaster preparedness. By incorporating temperature effects and quantifying prediction uncertainties, the study addresses key limitations in existing methodologies. Future work will expand this framework to assess spatiotemporal rainfall variability in Bangladesh and explore advanced machine learning approaches to simultaneously model the bulk and tail of rainfall distributions.

## Introduction

Bangladesh (Latitude: $$23.6850^\circ \text {N}$$, Longitude: $$90.3563^\circ \text {E}$$), a least developed South Asian country, is highly vulnerable to climate change due to its geographical location, low-lying flat topography, low-lying coastal areas, high population density and dependence on agriculture. Extreme rainfalls and the resulting intense floods are annual disasters during the monsoon season, causing damage to homes, critical infrastructures, and agricultural activities, and affecting broadly various aspects of society, including the economy, water resources, transportation networks, and public health (Ahmed 2015; Fahad et al. 2024; Sarker and Rashid 2013; Ahmed 2015; Habib et al. 2023; Fahad et al. 2024) in Bangladesh. Therefore, understanding and predicting extreme rainfall, the main cause of intense floods, is essential, particularly in sectors such as water resource management, flood risk assessment, agriculture, and urban planning. From 1971 to 2014, in Bangladesh, the total economic losses of 78 floods amounted to approximately $12.2 billion (Kabir and Hossen 2019). According to a report by the Asian Development Bank, in 2014 alone, flood-related damage cost the Bangladeshi economy approximately $2.2 billion, equivalent to 1.5% of the country’s GDP (Ozaki 2016). An estimated cost of 2022 floods is $1.0 billion (Letsch et al. 2023).

Recent studies by Basher et al. (2020); Das (2021); Dastagir (2015); Chowdhury et al. (2019); Mohsenipour et al. (2020) have used different geostatistical models and estimation methods that project a significant increase in extreme rainfall in northeast Bangladesh due to climate change, particularly during the premonsoon season, leading to an increased risk of flash floods in the future, emphasizing the critical role of climate modeling in understanding these trends and promoting further research in the country. Another study found that temperatures have generally increased in Bangladesh, particularly during the summer, rainy, and autumn seasons, while annual rainfall trends have decreased, with wetter rainy seasons and drier winters, indicating more extreme seasonal contrasts during 1989–2018 (Das and Zhang 2021). Bari et al. (2016) analyzes 30 years of rainfall data in northern Bangladesh, revealing increasing trends in pre-monsoon and monsoon rainfall along with decreasing post-monsoon and winter rainfall. Habib et al. (2023) investigates the effect of low-level wind conditions leading to extreme rainfall in Chattogram, and the findings indicate a crucial role for moisture-laden westerlies in causing extreme rainfall.

Several studies analyze and investigate the intensity and trends of extreme rainfall or precipitation in non-stationary time series events using frequentist and Bayesian versions of extreme value (EV) and generalized EV models to predict future events and quantify uncertainties for those events in different countries, particularly in the USA, UK, Europe, Brazil, and India (Agilan and Umamahesh 2016, 2017; Gabriel Anzolin et al. 2023; Anzolin et al. 2024; Lee et al. 2020; Moustakis et al. 2021; Prosdocimi et al. 2014; Prosdocimi and Kjeldsen 2021; Ragno et al. 2018, 2019; Serago and Vogel 2018). A number of other studies have also contributed significantly to our understanding of modeling extreme events with a focus on the challenges posed by non-stationarity (Ouarda et al. 2019, 2020; Cheng and AghaKouchak 2014; Ouarda and Charron 2018; Thiombiano et al. 2017; Villarini et al. 2009; Hundecha et al. 2008; El Adlouni et al 2007). These studies also investigate the comparative advantages of non-stationarity in series for disaster preparedness and policymaking by governments.

Although various studies have been employed to analyze extreme rainfall in Bangladesh, most existing studies are simple explanatory trend analyses or are based on simplistic normality assumptions, which may not capture the complexity of the data (Abdullah et al. 2022; Shahid 2011; Chowdhury et al. 2019; Alam et al. 2018; Rimi et al. 2022). Recently, in a limited and regional periphery, there has been increasing interest in using advanced statistical and machine learning models (e.g. ${\mathcal {L}}$-Moments, quantile regression, random forest (RF) and extreme learning machine (ELM)) to evaluate extreme rainfall in Bangladesh (Rahman et al. 2013; Mohsenipour et al. 2020; Das 2021; Yaseen et al. 2021). Studies are yet to address the non-stationary behavior of the tail of the extreme rainfall distribution and uncertainty quantification in the prediction of extreme rainfall in Bangladesh.

In this study, our primary goal is to explore the avenue of non-stationary tail distribution of extreme rainfall in the Dhaka and Chattogram regions and the corresponding uncertainty quantification. To overcome the seasonality of the data, we mainly focus on extreme rainfall in the summer monsoon season, spanning from May to October each year. In particular, we model extreme rainfall events during summer monsoons in two major cities in Bangladesh, namely Dhaka, and Chattogram, using nonstationary extreme value models by incorporating atmospheric dry bulb temperatures as a covariate. We also predict the timing and likelihood of extreme rainfall events. However, all predictions come with associated uncertainties. We quantify uncertainties in our predictions using the standard delta method and the parametric bootstrap approach.

The remainder of this paper is organized as follows. In Sect. 2, we describe the data employed in our analysis. Section 3 presents the proposed methodology for modeling extreme rainfall. The descriptions of the selected models are described in Sect. 4. The results of modeling and probabilistic forecasting are reported in Sect. 5. Section 6 concludes the paper.

## Data

**Fig. 1:**
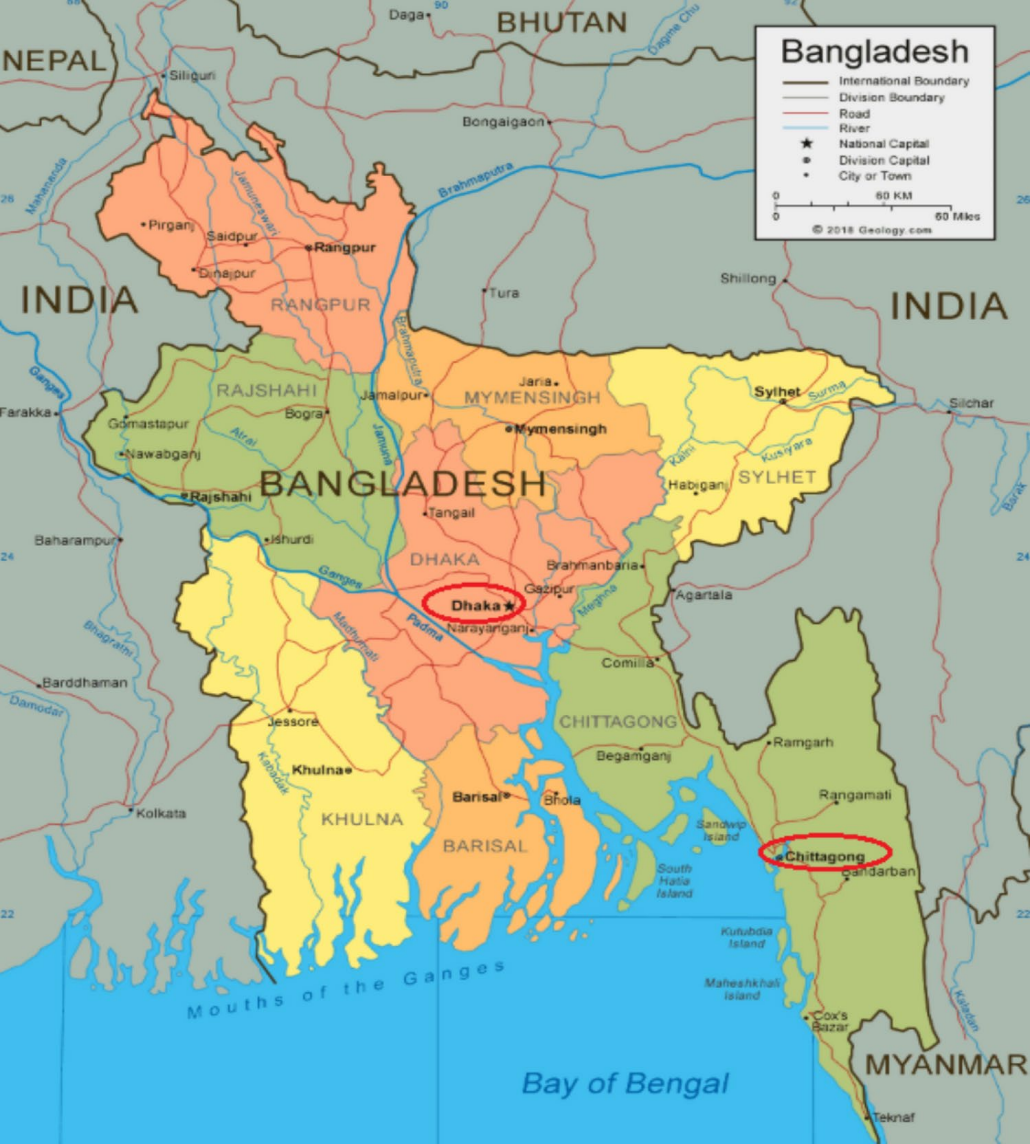
Study Areas in Bangladesh: Dhaka and Chattogram (pointed using red ovals). Different colors in map represent administrative divisions

This study utilizes daily rainfall measured in millimeters (mm) and dry bulb temperature measured in degree Celsius ($^\circ {\text{C}}$) for two major cities in Bangladesh: Dhaka (Coordinates: $23^\circ 45^\prime 50^{\prime \prime}{\text{N}} \ \ 90^\circ 23^\prime 20^{\prime \prime}{\text{E}}$) and Chattogram Coordinates: $22^\circ 20^\prime 06^{\prime \prime}{\text{N}} \ \ 91^\circ 49^\prime 57^{\prime \prime}{\text{E}}$). The geographic locations of these two cities are circled and presented in Fig. 1. The data cover a significant time period, with 26 years of daily data from 1990 to 2015 for Dhaka (recorded at Agargaon station) and 17 years of daily data from 1999 to 2015 for Chattogram (recorded at Ambagan station). Here, we assume that the distributions of Chattogram’s rainfall and temperature in 1990–1998 are the same as their distributions in 1999–2015, which reduces the potential biases in the model outputs for different temporal coverages in the two cities. The study focuses on the monsoon or rainy season, spanning from May to October each year. The data for both Dhaka and Chattogram are obtained from the Bangladesh Meteorological Department (BMD 2022). Table 1 provides an overview of the datasets that are used in this study. To model and analyze extreme rainfall events, the study incorporates dry bulb temperature as an exogenous variable. To address uncertainty due to inconsistencies and gaps, we have identified erroneous entries by cross-referencing with historical records. In the necessary situation, we have applied statistical techniques to detect outliers.

**Table 1:** Overview of the datasets

Weather station	Period	Data source	Variables
Dhaka	1990–2015	Bangladesh Meteorological Department	Rainfall, Temperature
Chattogram	1999–2015	Bangladesh Meteorological Department	Rainfall, Temperature

**Fig. 2:**
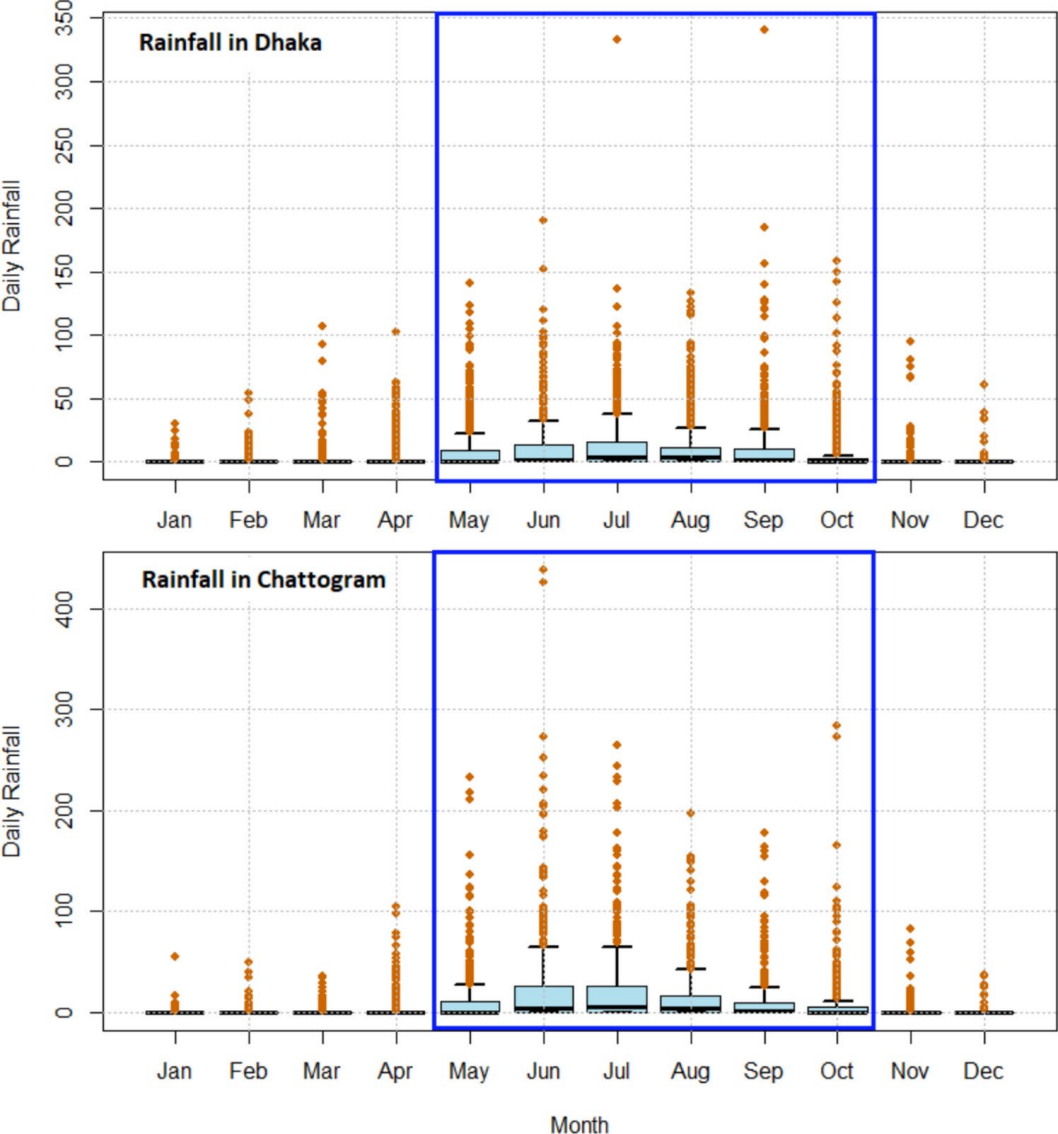
Daily Rainfall pattern in 12 months in Dhaka (top panel) and Chattogram (bottom panel)

Figure 2 shows insights into the monthly rainfall patterns over the years. The top panel showcases boxplots of the daily rainfall recorded in a 24-hour period in different months over a 26-year period in Dhaka, while the bottom panel represents the same information but for the 17-year period in Chattogram. We can see both cities broadly have two long seasons, i.e., (i) long monsoon season, spanning from May to October (blue rectangles in Fig. 2), and (ii) non-monsoon season spanning from November to April. In extreme value modeling, one of the widely adopted techniques to deal with data that vary seasonally is to partition the data into seasons within which data are assumed to be homogeneous, and perform a separate modeling on each season (Smith 1989; Coles and Walshaw 1994). In this study, for both Dhaka and Chattogram, we focus on evaluating the risk of severe rainfall during the long monsoon season only, particularly between May and October.

## Methods

Statistical methods and techniques are mainly focused on the center of a distribution, e,g., mean or median. However, in extreme value analysis, the interest is centered on the tail of the distribution. Extreme Value Theory (EVT) deals with the extreme deviations from the center of probability distributions and assesses the probability of more extreme events than a certain large value.

Let $Y_1, Y_2,..., Y_n$ be a sequence of independent random variables, with a common distribution function *G*. We define

1 $$\begin{aligned} M_n=\max \left\{ Y_1, Y_2, \cdots, Y_n\right\}, \end{aligned}$$

where $Y_i$, $i=1,2, \cdots, n$, represents the value of a process that is measured on a regular time scale. $M_n$ is the maximum of the process over *n* time units (i.e.,*block*) of the observation. If there exist sequences of constants $\{a_n >0\}$ and $\{b_n\}$ then

$$\begin{aligned} Pr\left\{ \left( M_n - b_n \right) /a_n \le z\right\} \rightarrow G(z) \end{aligned}$$

as $n \rightarrow \infty$, where *G* belongs to the family of Generalized Extreme Value (GEV) distribution with the following form

2 $$\begin{aligned} G(z)=\exp \left\{ -\left[ 1 + \xi \left( \frac{z-\mu }{\sigma }\right) ^{-\frac{1}{\xi }}\right] \right\}, \end{aligned}$$

which is defined on the set $\left\{ z: 1 + \xi (z-\mu )/ \sigma >0\right\}$, where the location parameter $(\mu )$, scale parameter $(\sigma )$ and shape parameter $(\xi )$ satisfy respectively, $-\infty<\mu <\infty$, $\sigma >0$ and $-\infty<\xi <\infty$ (Ouarda et al. 2020; Cheng and AghaKouchak 2014; Fisher and Tippett 1928; Coles 2001; Dey et al. 2019).

This method is also known as the *block maxima* approach. The maximum likelihood estimation (MLE) can be used to estimate the GEV model parameters (Coles 2001). The *blocks* are commonly selected with the time period of one year, in which case *n* is the number of observations in a year, where the block maxima are the annual maxima. The extreme quantile of the annual maximum distribution can be obtained in terms of the *r*-*year return level*, which is the level expected to be exceeded once every *r* years. The *r*-year return level can be derived from Eq. (2) as

3 $$\begin{aligned} z_r=\mu + {\sigma \over \xi } \left[ {\left( -\log \left( 1- r^{-1}\right) \right) ^{-\xi }}-1\right], \end{aligned}$$

for $\xi \ne 0$. We can also obtain the probability of an extreme event (*z*) as

4 $$\begin{aligned} P\left( Z>z\right) = 1 - \exp \left\{ -\left( 1 - \xi \left( \frac{\mu -z}{\sigma }\right) ^{-\frac{1}{\xi }}\right) \right\}. \end{aligned}$$

A non-stationary process has characteristics that change systematically through time due to many reasons, e.g., time trend, seasonal trend, covariate relationship, etc. A non-stationary extreme value distribution function (Ouarda et al. 2019; Hundecha et al. 2008) can be written as

5 $$\begin{aligned} F\left( x;\mu (t),\sigma (t),\xi (t)\right)= & \exp \left\{ -\Big [1+\xi (t) \Big (\frac{y-\mu (t)}{\sigma (t)}\Big )^{-\frac{1}{\xi (t)}}\Big ]\right\}. \end{aligned}$$

A non-stationarity can be introduced by expressing one or more of the GEV parameters with a function of time or/and other covariates, e.g., $\mu (t)$, $\sigma (t)$, $\xi (t)$. Here time *t* denotes the year or month or season over which the maximum is chosen. For example, one might consider a nonstationary GEV model with a time trend and covariate relationship in the location parameter as $\mu (t) = \beta _0 + \beta _1 t + \beta _2 S_{t}$, where *S* is a given covariate, the other GEV parameters remain fixed, i.e., $\sigma (t)=\sigma$ and $\xi (t)=\xi$ (Reiss and Thomas 2007; Dey and Das 2022).

We select appropriate non-stationarity extreme value models (i.e., covariates for model parameters) based on Akaike’s information criteria (AIC) (Akaike 1998) and the Bayesian information criterion (BIC) (Schwarz 1978). The AIC and BIC can be defined as $AIC =-2\ell + 2k, BIC = -2\ell + k \ln (n)$, respectively, where $\ell$ is the maximized log-likelihood function of the model and *k* is the number of parameters in the model. Both AIC and BIC penalize the log-likelihood function for the number of parameters estimated. AIC and BIC are measured on a relative (or interval) scale. An individual AIC or BIC value, by itself, is not interpretable due to the unknown constant (interval scale). AIC or BIC is only comparative, relative to other AIC values in the model set. The model with the smallest AIC and BIC is considered the “best” model. Sometimes, it is suggested that if the difference between two AIC/BIC scores is greater than 2, then the model with the smaller AIC/BIC score is more supported. If the difference is less than 2, then both models are equally well supported (Burnham and Anderson 2002; Burnham et al. 2011; Berchtold 2010; Yin and Miller 2022). The quantile-quantile (Q-Q) plots and observed versus fitted density are popular techniques for model diagnostics.

We compute the *r*-year return level and the probability of an extreme event from the selected non-stationarity extreme value model similar to the stationary GEV model.

## Modeling extreme rainfall using block maxima approach

In Sect. 2 we describe the rainfall data in Dhaka and Chattogram. We find that in both cities the majority portions of total rainfall in a year occur during summer, from May to October. Therefore, in this study, we only consider the rain from May to October each year and model the extreme rainfall during this summer monsoon. We focus on the tail of the summer monsoon rainfall in Dhaka and Chattogram. To model the extreme rainfall in the summer monsoon in two major cities in Bangladesh, i.e., in Dhaka and Chattogram, we apply the GEV *block maxima* approach, where monsoon months are considered as blocks.

### Variable definitions

Suppose in a particular month the maximum rainfall *Y* (i.e., $M_n$ in Eq. 1) occurs on the day *l*, $l=1, 2, \ldots, n$, where *n* is the total number of days in that month. Villafuerte and Matsumoto (2015); Ivancic and Shaw (2016); Sharma and Mujumdar (2019); Wang et al. (2017); Marra et al. (2024); Lepore et al. (2015); Lee et al. (2020a, 2020b); Roderick et al. (2020), and Magan et al. (2020) study the relationship between temperature and extreme rainfall. Sometimes the high rainfall on one day can result from lag temperatures (Sharma and Mujumdar 2019; Marra et al. 2024). In this study, to assess the effect of temperature on the monthly maximum rainfall, we consider the average temperature on the day *l*, i.e., $T_l$, as a covariate. We also consider lag days temperatures, i.e., $T_{l-k}$, as covariates. In addition, we choose the average and maximum of the last *k* day’s temperature ($T_{mean}$, and $T_{max}$, respectively) as potential covariates, where the maximum lag *k* is chosen experimentally.

We have 26 years of data for Dhaka, and 17 years of data for Chattogram, where for both cases each year contains 6 summer monsoon months. Therefore for Dhaka, $Y_t$ represents a vector of 156 monthly maximum rainfall records, where $t=1,2, \ldots, 156$. Similarly, for Chattogram, $Y_t$ represents a vector of 102 monthly maximum rainfall records, where $t=1,2, \ldots, 102$. These sample sizes are reasonable for using maximum likelihood estimation (Reiss and Thomas 2007; Coles 2001; Dey and Yan 2015; Papalexiou and Koutsoyiannis 2013).

**Fig. 3:**
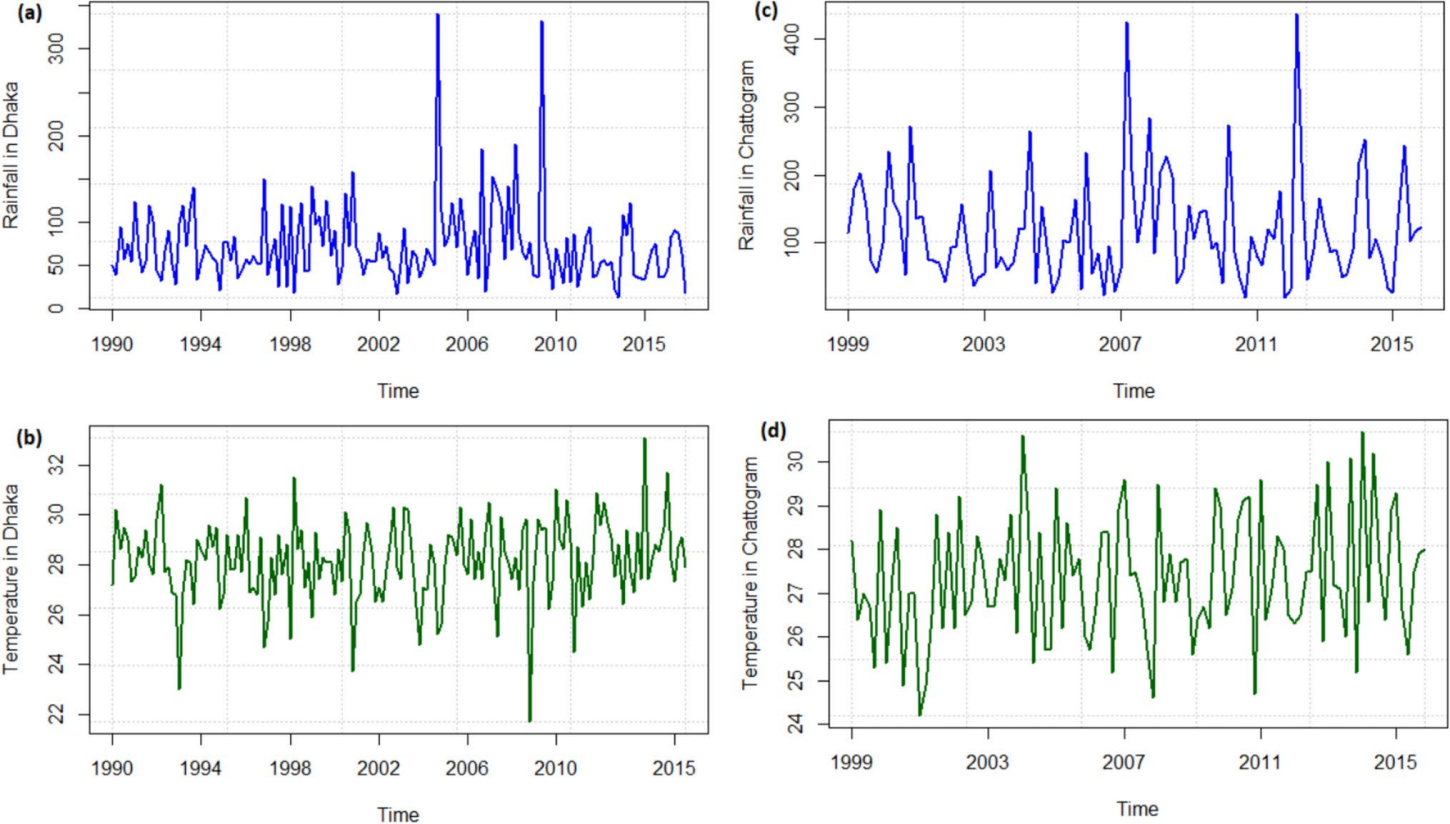
Observed monthly maximum 24-hours rainfall and 2-day lag temperature in Dhaka and Chattogram

Figure 3 presents time plots for monthly maximum 24-hour rainfall in Dhaka (Fig. 3a) and Chattogram (Fig. 3c). Here we only consider the data for the summer monsoon, i.e., from May to October each year. The figure also shows the dynamics of the lag temperatures. Figure 3b and d represent 2-day lag temperature of the monthly maximum rainfall in Dhaka and Chattogram, respectively. While no clear trends are evident in the time plots, the graphs reveal multiple coinciding opposite directional spikes, suggesting a potential negative relationship between maximum rainfall and lag temperature. One possible reason for this opposite relationship is that it has been raining for the last few days which decreases the temperature.

### Model selection and estimation

To select the best performing model we have conducted extensive experiments. We start with the stationary GEV *block maxima* model, where the monthly maximum rainfall in summer monsoon ($Y_t$) is considered to follow a generalized extreme value distribution with parameters $\mu$ (location), $\sigma$ (scale), and $\xi$ (shape), i.e., $Y_t \sim GEV(\mu, \sigma, \xi )$. We consider this stationary model as a base model and name this as Model 0.

To choose lag temperatures and their functions, e.g., the average and maximum of the last *k* day’s temperature, as covariates for the non-stationary extreme value models, we experimentally select the maximum lag $k=8$. In addition, we conduct a thorough experiment with a set of non-stationary extreme value models, $Y_t \sim GEV(\mu (t), \sigma (t), \xi (t)).$ We assess the different combinations of time trends and temperature effects on the monthly maximum rainfall in the summer monsoon. In the model fitting process, we consider the monthly maximum rainfall GEV models (stationary and non-stationary) in the summer monsoon with 6 months as a yearly maximum rainfall GEV model taking 6 largest values in each year (Coles 2001).

Table 2 represents the showcase of the best four models for the both city of Dhaka and Chattogram with their AIC and BIC values. For Dhaka, in Model 1 the location parameter depends linearly on lag temperature, and in Model 2 the location parameter depends linearly on the maximum of the last 8 days temperature. Finally, the location parameter of Model 3 for Dhaka depends on the mean of lag temperatures, and there is also a time trend in the log-scale parameter. We estimate the models with maximum likelihood estimation (MLE) methods. We find that, for Dhaka, Model 1 gives the best AIC and BIC values. Now, we focus on modeling extreme rainfall for the city of Chattogram. For Chattogram, we also find that Model 1, where the location parameter depends linearly on lag temperature and the log-scale parameter has a linear time trend, gives the best AIC value. Notice that for both Dhaka and Chattogram, Model 1 which contains a 2-day lag for temperature as a covariate gives the best AIC and BIC values compared to their competitors. Therefore, we select Model 1 s are our final models for both Dhaka and Chattogram.

**Table 2:** Different stationarity/non-stationary models with the corresponding AIC and BIC for Dhaka and Chattogram regions

	Model	Non-stationarity	AIC	BIC
Dhaka	Model 0 (Stationary)	$Y_t \sim GEV(\mu, \sigma, \xi )$	1551.154	1560.304
	Model 1	$Y_t \sim GEV(\mu (t), \sigma, \xi )$	1547.507	1559.707
		$$\mu (t)=\beta _0 + \beta _1 T^t_{l-2}$$		
	Model 2	$Y_t \sim GEV(\mu (t), \sigma, \xi )$	1553.107	1565.306
		$$\mu (t)=\beta _0 + \beta _1~T^t_{max}$$		
	Model 3	$Y_t \sim GEV(\mu (t), \sigma (t), \xi )$	1552.476	1567.726
		$$\mu (t)=\beta _0 + \beta _2~T^t_{mean}$$		
		log $\sigma (t)= \gamma _0 + \gamma _1 {t}$		
Chattogram	Model 0 (Stationary)	$Y_t \sim GEV(\mu, \sigma, \xi )$	1145.115	1152.99
	Model 1	$Y_t \sim GEV(\mu, \sigma (t), \xi )$	1141.614	1154.739
		$$\mu (t)=\beta _0 + \beta _1 T^t_{l-2}$$		
		log $\sigma (t)= \gamma _0 + \gamma _1 {t}$		
	Model 2	$Y_t \sim GEV(\mu (t), \sigma, \xi )$	1147.03	1157.53
		$$\mu (t)=\beta _0 + \beta _1~T^t_{max}$$		
	Model 3	$Y_t \sim GEV(\mu (t), \sigma (t), \xi )$	1146.81	1159.935
		$$\mu (t)=\beta _0 + \beta _2~T^t_{mean}$$		
		log $\sigma (t)= \gamma _0 + \gamma _1 {t}$		

First, we evaluate the diagnostic plots of Model 0 (stationary model). Figures 8 and 9 in Appendix A show the density plots and Q-Q plots for Dhaka and Chattogram, receptively. We observe that the density plots do not yield a good fit, and Q-Q plots show a deviation from the standard 45-degree line, particularly for Dhaka. Figure 10 represents rainfall return levels of Dhaka and Chattogram based on the corresponding stationary models. We find that the 95% confidence intervals of the return levels can not capture the two most extreme rainfall events in Dhaka. For Chattogram, the uncertainties of the return levels are very large (wider confidence intervals). Return levels of extreme rainfalls based on selected non-stationary models are discussed in Sect. 5.

**Fig. 4:**
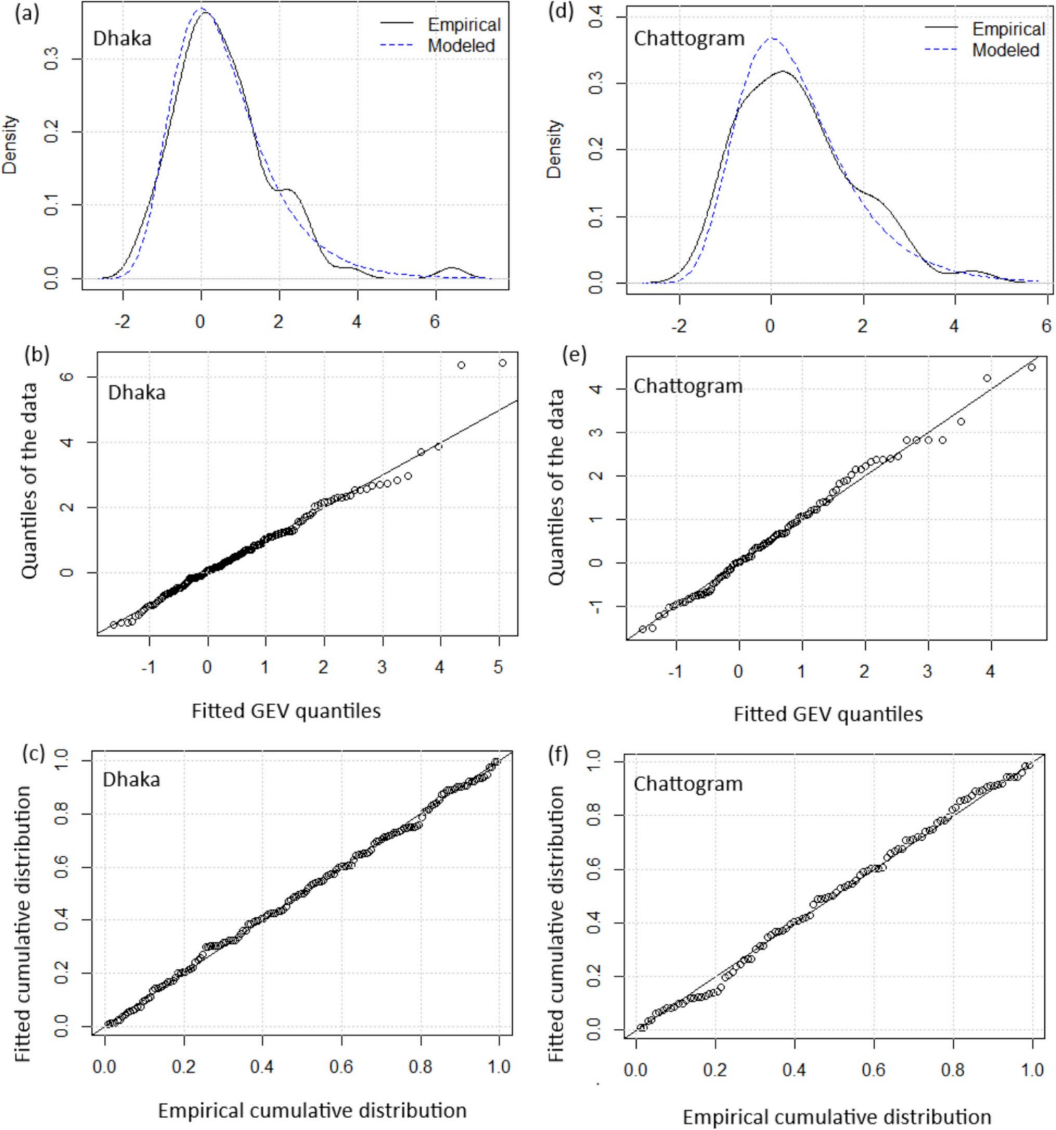
Goodness of fit plots for selected models for Dhaka and Chattogram

Figure 4 shows the diagnostic plots for the selected models for Dhaka and Chattogram (For both cases, Model 1). The density plots for Dhaka and Chattogram (Fig. 4a and d, respectively) yield that the selected models capture the variability of the monthly maximum rainfall in Dhaka and Chattogram during the summer monsoon. The Q-Q plot for Dhaka (Fig. 4c) appears to miss the very upper tail, in particular, the two most outlying observations, however, the Q-Q plot for Chattogram (Fig. 4e) represents a good fit (approximately 45-degree line). The empirical versus fitted cumulative distribution plots (Fig. 4c and f) also show good fits. Therefore, we can say that the selected GEV models are reasonable fits for modeling monthly maximum rainfall in Dhaka and Chattogram during the summer monsoon.

**Table 3:** Estimated parameters, standard error estimates are in the parentheses, with 95% confidence intervals of the selected GEV models (i.e., Model 1 s) for Dhaka and Chattogram

	Parameter	Estimate	$$L_{0.025} $$	$$ U_{0.975}$$
Dhaka	${\beta }_0$	135.230 (38.077)	60.601	209.859
	${\beta }_1$	− 2.923 (1.341)	− 5.551	− 0.295
	${\sigma }$	26.414 (1.822)	22.843	29.986
	${\xi }$	0.147 (0.061)	0.147	0.265
	$\beta _0$	306.486 (90.691)	128.734	484.239
Chattogram	$\beta _1$	− 8.286 (3.287)	− 14.729	− 1.845
	${\gamma }_0$	3.675 (0.192)	3.301	4.051
	${\gamma }_1$	0.003 (0.003)	− 0.003	0.009
	${\xi }$	0.202 (0.100)	0.006	0.398

Table 3 presents the estimated parameters with 95% confidence intervals for the selected models for Dhaka and Chattogram. It is noted that, for both Dhaka and Chattogram, lag temperatures show a negative relationship with rainfall. Using the estimated parameters we can write the fitted non-stationary GEV models for the extreme rainfall $Y_t$ during the summer monsoon in Dhaka and Chattogram as:

6 $$\begin{aligned} & \text {Dhaka - Model~1:}~~ {\hat{Y}}_t \sim GEV({\hat{\mu }} (t), {\hat{\sigma }}, {\hat{\xi }}),~\text {where}\nonumber \\ & {\hat{\mu }}(t) = 135.230 -2.923 T^t_{l-2}. \end{aligned}$$

7 $$\begin{aligned} & \text {Chattogram - Model~1:}~~ {\hat{Y}}_t \sim GEV({\hat{\mu }} (t), {\hat{\sigma }}(t), {\hat{\xi }}),~\text {where} \nonumber \\ & {\hat{\mu }}(t) = 306.486 -8.286 T^t_{l-2},\nonumber \\ & {\hat{\sigma }}(t) = \exp \left\{ 3.675 + 0.003 t \right\}. \end{aligned}$$

It is important to note that one of the primary concerns raised is the influence of small sample sizes on MLE when estimating the GEV distribution parameters. Studies such as Martins and Stedinger (2000) discuss the limitations of MLE and bias in shape parameter estimation. This is particularly relevant in cases where the GEV shape parameter is large, as observed for Chattogram. In addition, the quantile underestimation observed in Dhaka for low exceedance probabilities can be tied to the non-stationary behavior in the precipitation data. A study on African cities by Hossain et al. (2021) suggests that non-stationary models perform better under changing climatic conditions, whereas traditional stationary models like the GEV distribution can lead to misestimation of quantiles.

## Risk quantification

In this section, we evaluate the risk of extreme rainfall in Dhaka and Chattogram in the summer monsoon on the basis of return level and probability of extreme rainfall. We also measure the uncertainty of estimated return revel and probabilities of extremes based on standard delta methods and parametric bootstrap approach, respectively.

### Return level of extreme rainfall

We estimate the return level, i.e., the extreme quantile of rainfall which is expected to exceed on average once every *r* years, for Dhaka and Chattogram based on Eq. 3 as

$$\begin{aligned} \text {Dhaka:}~~{\hat{z}}_r={\hat{\mu }}(t) + {{\hat{\sigma }}\over {\hat{\xi }}} \big [{\big (-\log (1- r^{-1})\big )^{-{\hat{\xi }}}}-1\big ],\\ \text {Chattogram:}~~{\hat{z}}_r={\hat{\mu }}(t) + {{\hat{\sigma }}(t)\over {\hat{\xi }}} \big [{\big (-\log (1- r^{-1})\big )^{-{\hat{\xi }}}}-1\big ], \end{aligned}$$

where the nonstationary parameters for Dhaka and Chattogram, i.e., ${\hat{\mu }}(t)$ for Dhaka, and ${\hat{\mu }}(t)$ and ${\hat{\sigma }}(t)$ for Chattogram, are defined in Eqs. (6 and 7), respectively. The return level computation is similar to yearly return level computation with yearly maximum rainfall GEV model taking 6 largest values in each year (i.e., block).

For the city of Dhaka, return levels $z_r$ depend on the covariate lag temperature $T_{t-2}$, and for Chattogram, return levels $z_r$ depend on lag temperature $T_{t-2}$ and time trend *t*. We can compute rainfall return level, i.e., the estimate of the total 24-hour rainfall that is expected to exceed once every *r* years for specific values of *r* for certain values of the covariates.

Now we focus on the uncertainties associated with the estimated return levels in order to avoid misleading inferences. One main source of uncertainty in extreme value modeling comes from the uncertainties in the fitted model parameters (Wehner 2010; Das and Dey 2016). This uncertainty is usually measured by the standard error and confidence intervals. We compute a 95 percent confidence interval for the return level ${z_r}$ using the delta method (Doob 1935; Hoef 2012) under the assumption of the asymptotic normality of the estimate of ${z}_r$.

**Fig. 5:**
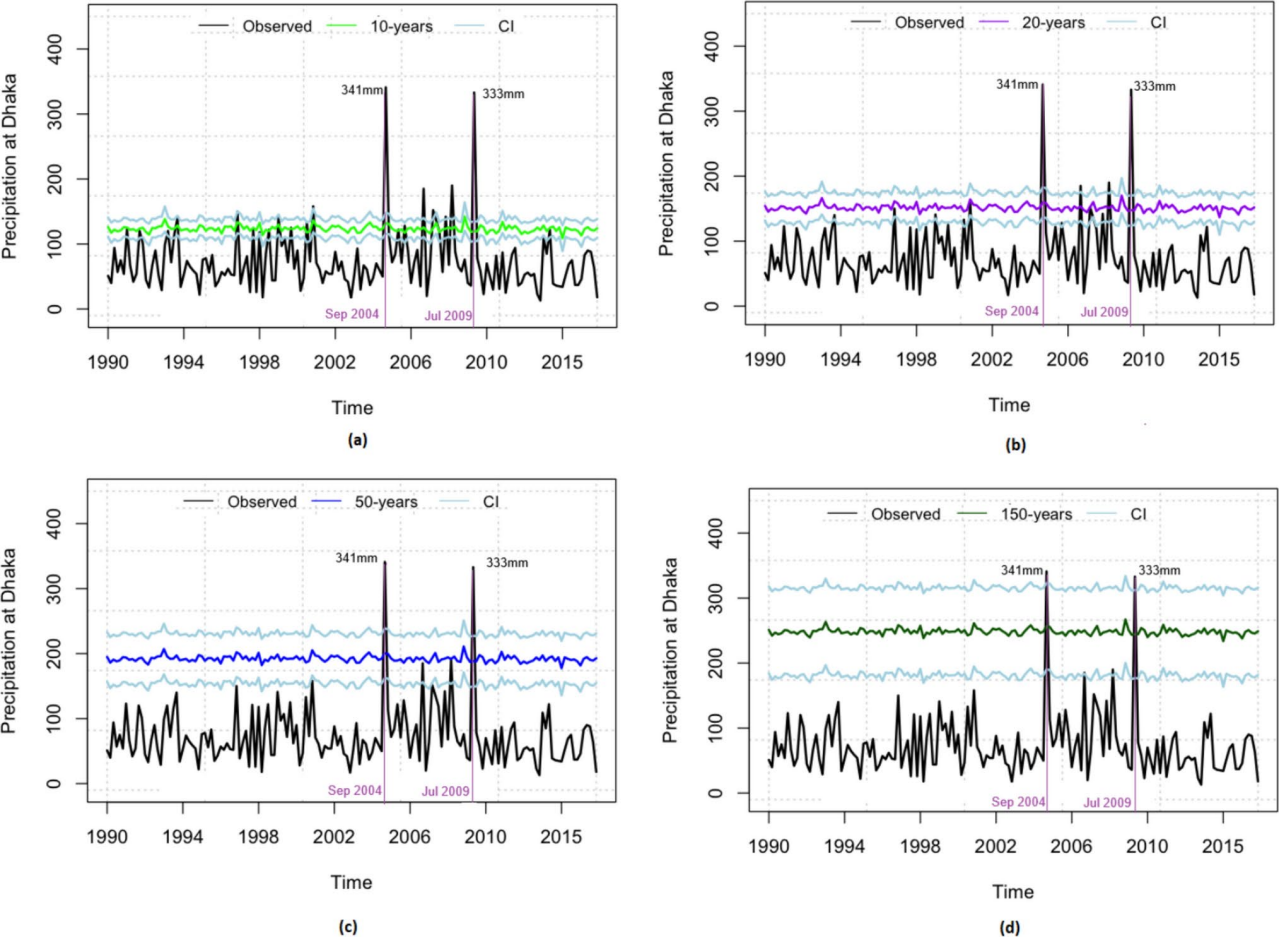
Rainfall return levels with 95% confidence intervals (CI) for different return periods, based on Model 1 for the city of Dhaka

Figure 5 represents 10, 20, 50, and 150 years of rainfall return levels with 95% confidence intervals for Dhaka correspond to the observed values of covariate $T_{t-2}$. Notice that an unusual 341 mm of rain was recorded in Dhaka over the 24-hour period in September 2004. This is the highest recorded rainfall for any month in the city of Dhaka for a 24-hour period (BMD 2022). And, 333 mm of rainfall was recorded in a 24-hour period in July 2009, which is the highest rainfall ever recorded in a 24-hour period in July in Dhaka. From the fitted model we find that the upper bound of the 150-year rainfall return level approximately capture the 341 mm of rain in September 2004 and 333 mm of rain in July 2009. That is, the 95% upper confidence bound of the 150-year return level of the 24-hour rainfall can be as high as 341 mm and 333 mm, given the corresponding covariates.

**Fig. 6:**
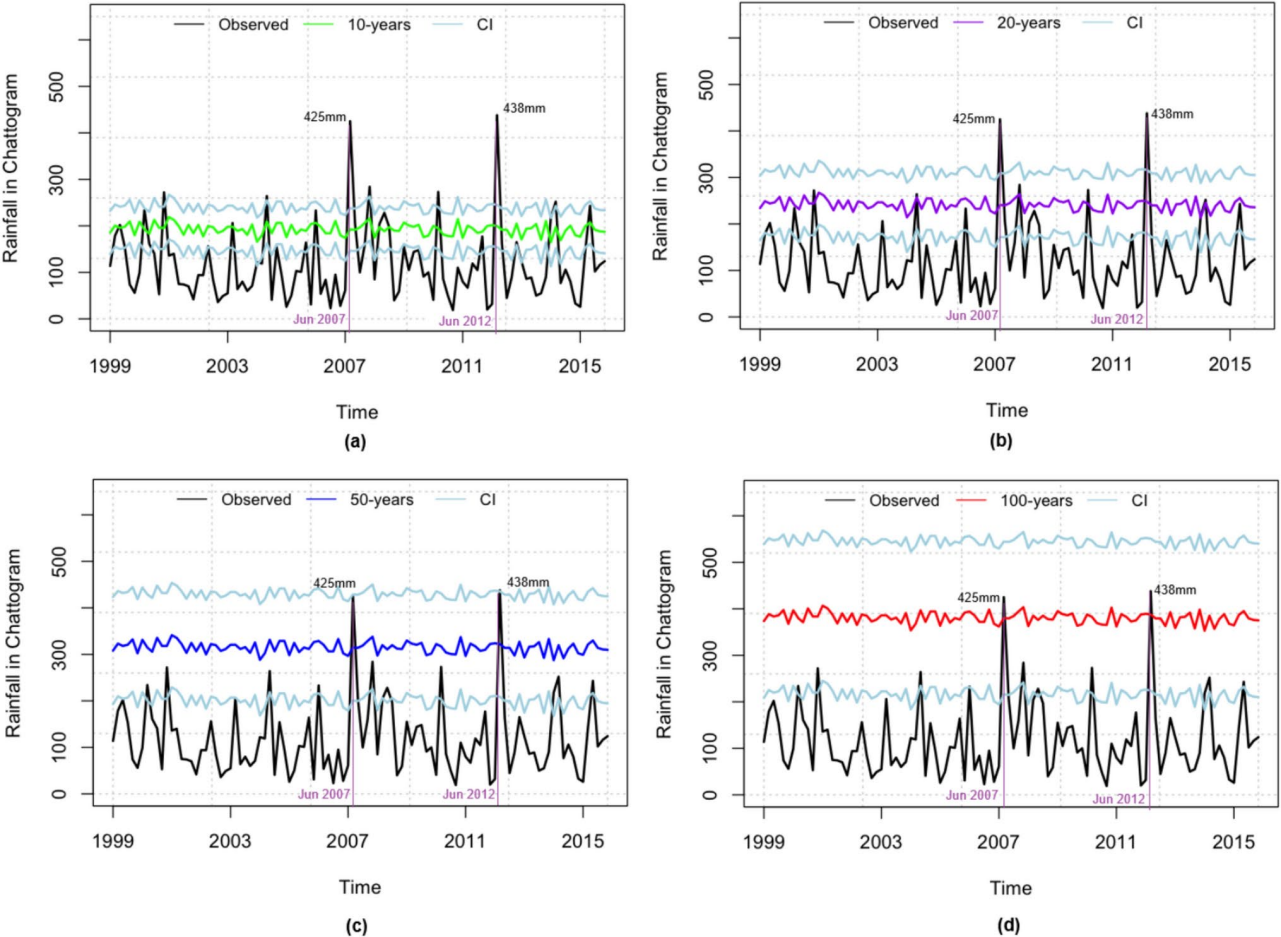
Rainfall return levels with 95% confidence intervals (CI) for different return periods, based on Model 1 for the city of Chattogram

Now we turn our analysis to evaluate the risk of extreme rainfall in Chattogram. Figure 6 shows return levels of extreme rainfall in Chattogram for 10, 20, 50, and 100 years return periods with corresponding 95% confidence intervals. It is obvious that Chattogram gets substantially heavier rainfalls than Dhaka, especially because of its geographic location. We find that, in Chattogram, a record 438 mm of rain was recorded over the 24-hour period in June 2012, and a total of 425 mm of rain was recorded in June 2007. From the fitted model, we find that the 95% upper confidence bound of the 50-year rainfall return level for Chattogram can be as high as 438 mm and 425 mm, given the corresponding covariates.

### Probability of extreme rainfall

We evaluate the probability of an extreme rainfall event, for Dhaka and Chattogram based on Eq. (4) as

$$ \begin{gathered} {\text{Dhaka:}}~~\hat{P}\left( {Z > z} \right) \hfill \\ \quad \quad \quad = 1 - \exp \left\{ { - \left( {1 - \hat{\xi }\left( {\frac{{\hat{\mu }(t) - z}}{{\hat{\sigma }}}} \right)^{{ - \frac{1}{{\hat{\xi }}}}} } \right)} \right\}, \hfill \\ {\text{Chattogram:}}~~~~\hat{P}\left( {Z > z} \right) \hfill \\ \quad \quad \quad = 1 - \exp \left\{ { - \left( {1 - \hat{\xi }\left( {\frac{{\hat{\mu }(t) - z}}{{\hat{\sigma }}}(t)} \right)^{{ - \frac{1}{{\hat{\xi }}}}} } \right)} \right\}, \hfill \\ \end{gathered} $$

where the nonstationary parameters for Dhaka and Chattogram are defined in Eqs. (6 and 7), respectively. Table 4 shows the risk of extreme daily rainfall in Dhaka and Chattogram in terms of probability. For example, the probability of observing a 150 mm 24-hour rainfall total or greater in Dhaka is 0.052; however, this probability is 0.190 in Chattogram. We find that for all the extreme quantiles the risks of extreme rainfalls in Chattogram are considerably higher than they are in Dhaka.

**Table 4:** Probability of exceeding extreme quantiles of rainfalls

Probability, ${\hat{P}}\left( Z>z\right)$		
Extreme quantiles (z)	Dhaka	Chattogram
150	0.052	0.190
200	0.017	0.087
250	0.007	0.043
300	0.003	0.023
350	0.001	0.013

We quantify the uncertainties in the probabilities of extreme rainfalls presented in Table 4 by confidence intervals. We obtain confidence intervals of the probabilities using a parametric bootstrap process. We briefly describe the parametric bootstrap in the following steps.

- *Step 1:* Let *G* be the selected GEV model of a particular city, and ${\hat{G}}$ be the corresponding fitted model.
- *Step 2:* Simulate a new random sample of size equal to the length of the original data from ${\hat{G}}$, i.e., GEV model with estimated parameters.
- *Step 3:* Using this new sample estimate *G*. We denote this new fitted model as $\hat{G^*}$. Using $\hat{G^*}$ obtain probabilities of extreme rainfall for specific *z* values based on Eq. (4).

Repeat Steps 2–3 2000 times. From these 2000 sets of estimated probability values for a particular *z*, we compute a 95% confidence interval based on the percentile method described in (Davison and Hinkley 1997; Dey et al. 2024).

**Fig. 7:**
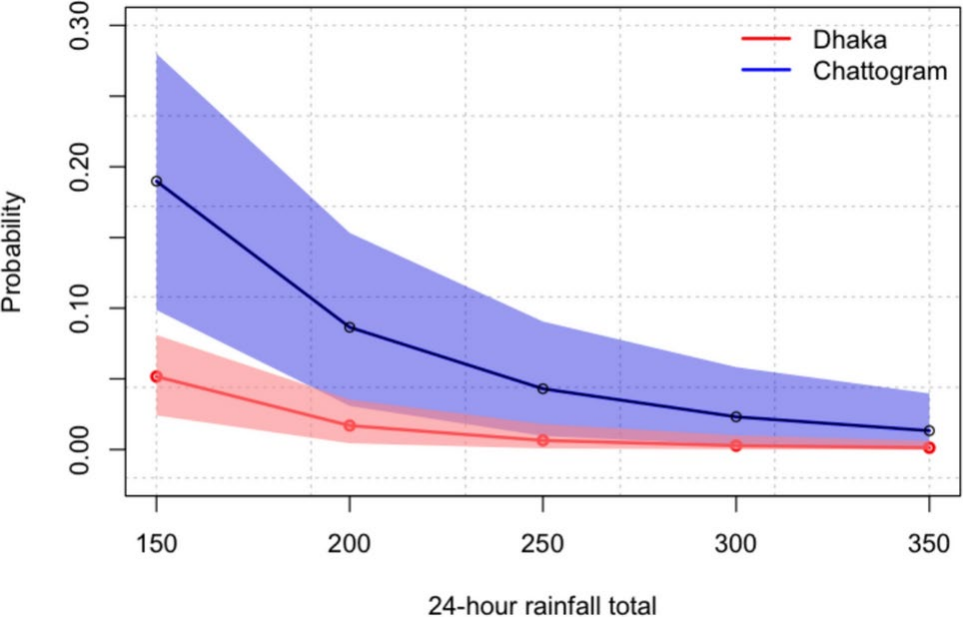
Estimated probability of extreme rainfalls with $95\%$ confidence intervals for Dhaka and Chattogram

**Fig. 8:**
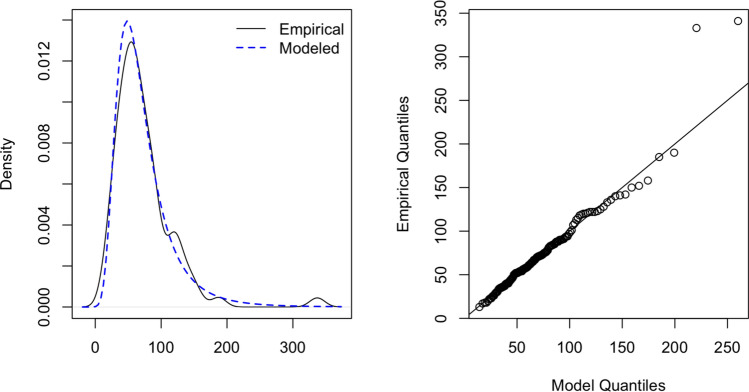
Goodness of fit of stationary model (Model 0) for Dhaka

**Fig. 9:**
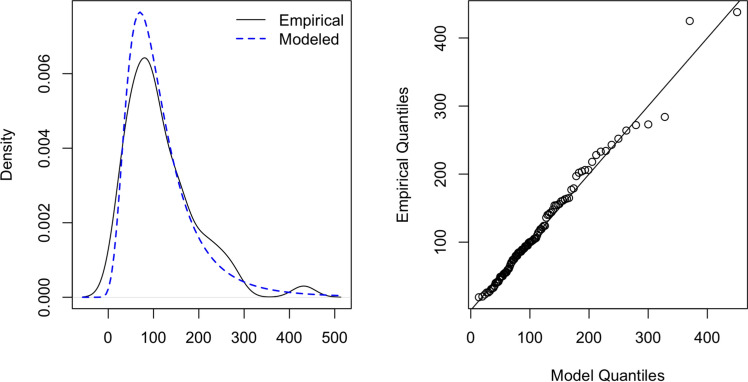
Goodness of fit of stationary model (Model 0) for Chattogram

**Fig. 10:**
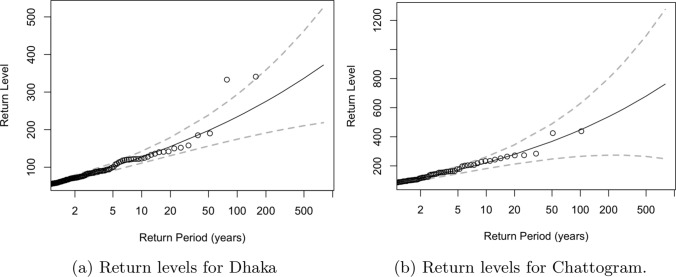
Rainfall return levels of Dhaka and Chattogram City for different return periods, based on stationary models (Model 0)

The results presented in Table 4 reveal that the probability of extreme rainfall, such as 150 mm in a 24-hour period, is significantly higher in Chattogram (0.190) compared to Dhaka (0.052), with probabilities decreasing for higher thresholds but consistently remaining greater in Chattogram. Moreover, uncertainty analysis displayed in Fig. 7 using a parametric bootstrap approach highlights wider 95% confidence intervals for Chattogram, indicating greater variability in predictions. These results emphasize Chattogram’s heightened vulnerability to extreme rainfall, necessitating enhanced flood preparedness, improved drainage systems, and climate-resilient infrastructure. In contrast, Dhaka faces relatively lower risks but still requires targeted adaptation strategies. The nonstationary GEV models effectively capture the dynamic nature of extreme rainfall, supported by atmospheric temperature as a covariate, offering reliable tools for probabilistic forecasting. Policymakers and urban planners can use these insights to inform disaster management and regional development plans, particularly in Chattogram. The study underscores the importance of addressing data gaps and refining models to reduce prediction uncertainties. Additionally, the methodological framework can be extended to other regions in Bangladesh, supporting a comprehensive national risk assessment, and future research could explore advanced models and alternative covariates to enhance predictive accuracy.

## Conclusion

In this paper, we focus on modeling and understanding extreme rainfall patterns in two major cities, Dhaka and Chattogram, within the broader context of Bangladesh. In our analysis, we apply monthly rainfall data for Dhaka and Chattogram for the periods of 1990– 2015 and 1999– 2015, respectively. Employing various versions of the nonstationary GEV models, we selected the most suitable models for Dhaka and Chattogram based on model selection criteria AIC, BIC, and goodness of fit plots. We estimate the selected GEV model parameters using maximum likelihood estimation. The models are then used to predict the quantum, timing, and likelihood of extreme rainfall events in Dhaka and Chattogram. The results offer valuable insights about extreme rainfall and its relationship with atmospheric dry bulb temperature. Our study finds that the likelihood of extreme rainfall in Chattogram is substantially larger than in Dhaka. The insights gained from this study can guide the management of agricultural activities, water resource management, flood risk assessment, urban planning, and disaster preparedness in Bangladesh.

One key limitation of the proposed modeling is that though the GEV model is a popular choice for modeling the tails of random variables and has been adopted in the context of modeling extreme rainfall, it only focuses on block maxima and does not provide any model for the bulk of the data. Therefore, a direction for future work will be to combine GEV models with machine learning/ deep learning models in a mixture model, which evaluates the tail as well as the bulk of the data (MacDonald et al. 2011; Wang and Gao 2023; Qi and Majda 2020; Nicola Gnecco and Engelke 2024; Pasche and Engelke 2024). A second limitation is that the model parameters that change with time and other covariates increase the estimation variance, especially when interest is in low exceedance probabilities. This concern has been expressed in literature, namely, in the studies by Emmanouil et al. (2022); Bador et al. (2018) and Mamalakis et al. (2017). The third limitation of the proposed modeling is that the current setting of GEV modeling does not support validating the proposed models over historical periods. Future work may also enhance the current analysis by fitting different GEV models over different historical periods. Finally, in this paper, we only focus on extreme rainfall in two major cities in Bangladesh due to the context, interest, and goal of the study. In the future, we will also expand our proposed framework to assess spatio-temporal variability and trends of extreme rainfalls throughout Bangladesh. By addressing these areas, future research can enhance the accuracy and applicability of extreme rainfall predictions, contributing to more resilient infrastructure and informed policymaking in climate-vulnerable regions like Bangladesh. The methods and insights presented here serve as a critical step toward understanding and mitigating the impacts of extreme rainfall in the face of climate change (Agilan and Umamahesh 2017; Anzolin et al. 2024; Lee et al. 2020; Moustakis et al. 2021; Prosdocimi and Kjeldsen 2021; Ragno et al. 2019; Serago and Vogel 2018).

## Data Availability

Data sets are available upon request.

## Appendix A: Stationary models

The diagnostic plots of Model 0, i.e., stationary model, for Dhaka and Chattogram are shown in Figs. 8 and  9, respectively. Figure 10 presents the return levels of extreme rainfalls in Dhaka and Chattogram based on stationary models.

## Code availability

All R codes are available upon request.
